# Identifying Clinical Terms in Medical Text Using Ontology-Guided Machine Learning

**DOI:** 10.2196/12596

**Published:** 2019-05-10

**Authors:** Aryan Arbabi, David R Adams, Sanja Fidler, Michael Brudno

**Affiliations:** 1 Department of Computer Science University of Toronto Toronto, ON Canada; 2 Centre for Computational Medicine Hospital for Sick Children Toronto, ON Canada; 3 Section on Human Biochemical Genetics National Human Genome Research Institute National Institutes of Health Bethesda, MD United States

**Keywords:** concept recognition, medical text mining, biomedical ontologies, machine learning, phenotyping, human phenotype ontology

## Abstract

**Background:**

Automatic recognition of medical concepts in unstructured text is an important component of many clinical and research applications, and its accuracy has a large impact on electronic health record analysis. The mining of medical concepts is complicated by the broad use of synonyms and nonstandard terms in medical documents.

**Objective:**

We present a machine learning model for concept recognition in large unstructured text, which optimizes the use of ontological structures and can identify previously unobserved synonyms for concepts in the ontology.

**Methods:**

We present a neural dictionary model that can be used to predict if a phrase is synonymous to a concept in a reference ontology. Our model, called the Neural Concept Recognizer (NCR), uses a convolutional neural network to encode input phrases and then rank medical concepts based on the similarity in that space. It uses the hierarchical structure provided by the biomedical ontology as an implicit prior embedding to better learn embedding of various terms. We trained our model on two biomedical ontologies—the Human Phenotype Ontology (HPO) and Systematized Nomenclature of Medicine - Clinical Terms (SNOMED-CT).

**Results:**

We tested our model trained on HPO by using two different data sets: 288 annotated PubMed abstracts and 39 clinical reports. We achieved 1.7%-3% higher F1-scores than those for our strongest manually engineered rule-based baselines (*P*=.003). We also tested our model trained on the SNOMED-CT by using 2000 Intensive Care Unit discharge summaries from MIMIC (Multiparameter Intelligent Monitoring in Intensive Care) and achieved 0.9%-1.3% higher F1-scores than those of our baseline. The results of our experiments show high accuracy of our model as well as the value of using the taxonomy structure of the ontology in concept recognition.

**Conclusion:**

Most popular medical concept recognizers rely on rule-based models, which cannot generalize well to unseen synonyms. In addition, most machine learning methods typically require large corpora of annotated text that cover all classes of concepts, which can be extremely difficult to obtain for biomedical ontologies. Without relying on large-scale labeled training data or requiring any custom training, our model can be efficiently generalized to new synonyms and performs as well or better than state-of-the-art methods custom built for specific ontologies.

## Introduction

### Background

Automatic recognition of medical concepts in unstructured text is a key component of biomedical information retrieval systems. Its applications include analysis of unstructured text in electronic health records (EHR) [1-3] and knowledge discovery from biomedical literature [4,5]. Many medical terminologies are structured as ontologies, adding relations between concepts and often including several synonyms for each term. One of the most widely used ontologies in the medical space is SNOMED-CT (Systematized Nomenclature of Medicine - Clinical Terms) [6], which provides structured relationships for over 300,000 medical concepts. SNOMED-CT is commonly used in EHR Systems to help summarize patient encounters and is fully integrated with the International Classification of Diseases - Ninth Revision (ICD-9) billing codes used in the United States and many other jurisdictions. The Human Phenotype Ontology (HPO) [7] is an arrangement of terms used to describe the visible manifestations, or phenotypes, of human genetic diseases. With ~12,000 terms, the HPO has become the standard ontology used in rare disease research and clinical genetics and has been adopted by the International Rare Diseases Research Consortium [8], ClinGen [9], and many other projects. Although both SNOMED-CT and the HPO provide a number of synonyms for each term, they usually miss many valid synonymous terms, as manually curating every term that refers to a concept is extremely difficult, if not impossible. For example, HPO provides four additional synonyms for the term “Renal neoplasm,” including “Kidney cancer” and “Renal tumors,” but it does not include synonyms such as “Renal cancer.” There are also many concepts in HPO, such as “Retinal neoplasm,” which are not given any synonyms in the ontology.

Many concept recognition and text annotation tools have been developed for biomedical text. Examples of popular tools for general purpose are the NCBO (National Center for Biomedical Ontology) annotator [10], OBO (Open Biological and Biomedical Ontologies) annotator [11], MetaMap [12], and Apache cTAKES (Clinical Text Analysis and Knowledge Extraction System) [13]. Other tools focusing on more specific domains have also been developed, such as BioLark [14] for automatic recognition of terms from the HPO and a tool by Lobo et al [15], which combines a machine learning approach with manual validation rules to detect HPO terms. Another example is the phenotype search tool provided by PhenoTips [16], which uses Apache Solr indexed on the HPO and has an extensive set of rule-based techniques to rank matching phenotypes for a query. Many of these systems consist of a pipeline of natural language processing components including a tokenizer, part-of-speech tagger, sentence boundary detector, and named entity recognizer (NER)/annotator. Generally, the NER/annotator component of these tools are based on text matching, dictionary look-ups, and rule-based methods, which usually require significant engineering effort and are often unable to handle novel synonyms that are not annotated in the ontology.

On the other hand, in the more general domain of natural language processing, many machine learning–based text classification and NER tools have been recently introduced [17-19]. Typically, these methods do not require manual rule-based engineering; however, they are dependent on large annotated text data for training. Popular among them is a model known as LSTM-CRF, in which long short-term memory (LSTM) [20], a variation of recurrent neural networks (RNNs) widely used for processing sequences such as text, is used to extract rich representations of the tokens in a sentence and is then followed by a conditional random field (CRF) [21] on top of these representations to recognize named entities.

Although these methods address a similar problem, they cannot be used directly for concept recognition, as the number of named entity classes is typically much lower than that of the concepts in medical ontologies. For instance, CoNLL-2003 [22], a data set widely used for evaluations of such methods, contains only four classes: locations, persons, organizations, and miscellaneous. As a result, these methods typically have a large number of training and test examples for each class, while in our setting, we are trying to recognize tens or hundreds of thousands of terms and may have only a few or even no examples of a specific term. Automatic creation of training data by exact match searching of the synonyms in a large corpus will not fully utilize synonyms that have no or low coverage in the data set, can bring bias by mislabeling valid out-of-ontology synonyms in the extracted snippets as negatives, and overfit to the context of the more frequent senses. Hence, in a setting where the training data does not fully cover all the classes, methods based on dictionary look-up might have some advantage, as they can identify a concept in a given text by simply matching it to a synonym available in their dictionary without requiring training data annotated with that concept.

In this paper, we develop a hybrid approach, called Neural Concept Recognizer (NCR), by introducing a neural dictionary model that learns to generalize to novel synonyms for concepts. Our model is trained on the information provided by the ontology, including the concept names, synonyms, and taxonomic relations between the concepts, and can be used to rank the concepts that a given phrase can match as a synonym. Our model consists of two main components: an encoder, which maps an input phrase to a vector representation, and an embedding table, which consists of the vector representations learned for the ontology concepts. The classification is performed based on the similarity between the phrase vector and the concept vectors. To allow for the use of our model to also detect concepts from longer texts, we scan the input text with fixed-size windows and report a phrase as matching a concept if it is above a threshold that is chosen from an appropriate validation data set.

Our work introduces a novel machine learning–based method for automatic concept recognition of medical terms in clinical text, and we have provided empirical results to demonstrate the accuracy of our methods in several settings. We trained our neural dictionary model on the HPO and used it to recognize concepts from 228 PubMed abstracts and 39 clinical reports of patients with rare genetic diseases. Additionally, we used a subset of concepts from SNOMED-CT that have matching terms in ICD-9 and experimented on 2000 Intensive Care Unit (ICU) discharge summaries from a Multiparameter Intelligent Monitoring in Intensive Care (MIMIC-III) data set [23]. In both settings, we trained our model solely on the ontology data and did not use the text corpora except to set the recognition sensitivity threshold and choose model hyperparameters from a small validation set. Although the main focus of this work is recognizing HPO and SNOMED-CT concepts, our method can be easily trained on other biomedical ontologies. The results of our experiments show the high accuracy of our model, which is on par with or better than hand-trained concept recognition methods. Our tool has already been used in two applications. It has been integrated with the PhenoTips tool to suggest concepts for clinical reports [16] and to automatically recognize occurrences of phenotypes in a clinical report for subsequent data visualization [24].

### Related Works

Recently, several machine learning methods have been used in biomedical NER or concept recognition. Habibi et al [25] trained the LSTM-CRF NER model, introduced by Lample et al [17], to recognize five entity classes of genes/proteins, chemicals, species, cell lines and diseases. They tested their model on several biomedical corpora and achieved better results than previous rule-based methods. In another work, Vani et al [26] introduced a novel RNN–based model and showed its efficiency on predicting ICD-9 codes in clinical notes. Both of these methods require a training corpus annotated with the concepts (loosely annotated in the case of Vani et al [26]).

Curating such an annotated corpus is more difficult for typical biomedical ontologies, as the corpus has to cover thousands of classes. For example, the HPO contains 11,442 concepts (classes), while, to the best of our knowledge, the only publicly available corpus hand annotated with HPO concepts [14] contains 228 PubMed abstracts with only 607 unique annotations that are not an exact match of a concept name or a synonym. Thus, training a method to recognize the presence of concepts in biomedical text requires a different approach when there is a large number of concepts.

The concepts in an ontology often have a hierarchical structure (ie, a taxonomy), which can be utilized in representation learning. Hierarchies have been utilized in several recent machine learning approaches. Deng et al [27] proposed a CRF-based method for image classification that takes into account inheritance and exclusion relations between the labels. Their CRF model transfers knowledge between classes by summing the weights along the hierarchy, leading to improved performance. Vendrov et al [28] introduced the order-embedding penalty to learn representations of hierarchical entities and used it for image caption retrieval tasks. Gaussian embeddings were introduced by Neelakantan et al [29] and learn a high-dimensional Gaussian distribution that can model entailment instead of single point vectors. Most recently, Nickel et al [30] showed that learning representations in a hyperbolic space can improve performance for hierarchical representations.

## Methods

In this section, we first describe the neural dictionary model that computes the likelihood that a given phrase matches each concept from an ontology, and then demonstrate how to apply the model to larger text fragments such as a full sentence, which may have multiple (or no) terms.

### Overview of the Neural Dictionary Model

**Figure 1:**
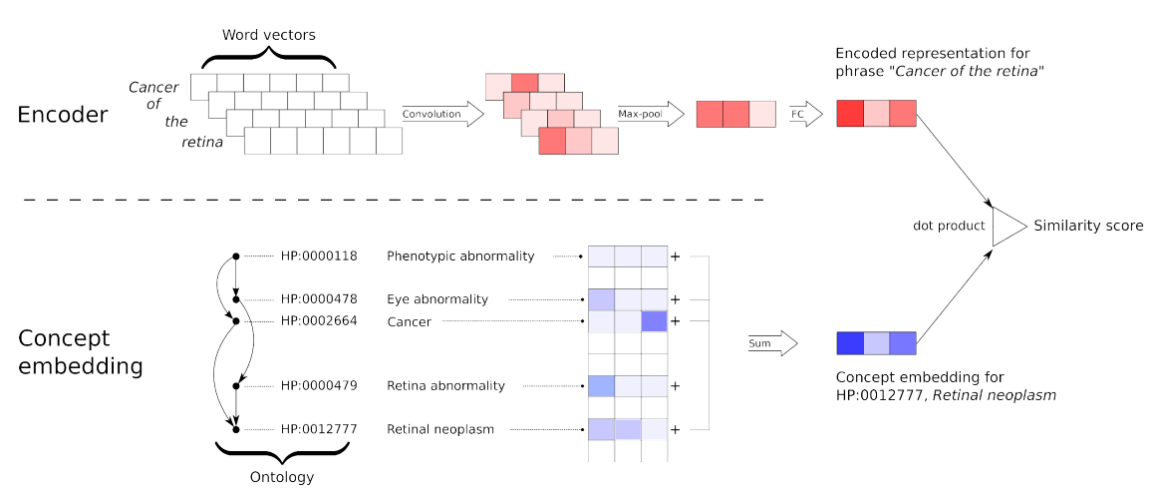
Architecture of the neural dictionary model. The encoder is shown at the top, and the procedure for computing the embedding for a concept is illustrated at the bottom. Encoder: a query phrase is first represented by its word vectors, which are then projected by a convolution layer into a new space. Then, a max-over-time pooling layer is used to aggregate the set of vectors into a single one. Thereafter, a fully connected layer maps this vector into the final representation of the phrase. Concept embedding: a matrix of raw embeddings is learned, where each row represents one concept. The final embedding of a concept is retrieved by summing the raw embeddings for that concept and all of its ancestors in the ontology. FC: fully connected.

The neural dictionary model receives a word or a phrase as input and finds the probability of the concepts in the ontology matching it. The model consists of a text encoder, which is a neural network that maps the query phrase into vector representation, and an embedding matrix with rows corresponding to the ontology concepts (Figure 1). We use the dot product of the query vector and a concept vector as the measure of similarity.

#### Encoder

We use word embeddings to represent the input words learned in a pre-processing step by running fastText [31] on publicly available MEDLINE/PubMed abstracts. The goal of this unsupervised step is to map semantically similar words (eg, synonyms) to close vectors. We selected fastText for this task primarily because it takes into account the subword information, which is important in the medical domain where there are many semantically close words with slight morphologic variations.

Inspired by the work of Kim et al [32], our encoder projects these word vectors into another space using a convolution neural network. We have used a much simpler network, consisting of a single convolution layer, with a filter size of one word. Although our choice of filter size has the disadvantage of losing the word order information, in our settings, this was outweighed by the benefit of having fewer network parameters to learn. We also tried other types of encoders such as different variations of LSTMs and small variants of attention-based encoders [33]. However, given the small amount of training data available, simpler encoders were more effective.

After the first layer of projection, the output vectors were aggregated into a single vector (*v*) using a max-over-time pooling operation, as shown in the following equation *v*=max_t_{ELU(*Wx^(t)^*+*b*)}, where *x^(t)^* is the word vector for the *t*th word in the phrase; *W* and *b* are the weight matrix and the bias vector of the convolution filter, respectively; and ELU [34] is the activation function we used in the convolution layer. It should also be noted that the max operation used in the equation above is an element-wise operation that takes the maximum value of each feature across projected word vectors. Finally, a fully connected layer with the weights *U* was applied on *v*, followed by a ReLU (rectified linear unit) activation and *l2* normalization. The result *e* was used as the encoded vector representation of the phrase: 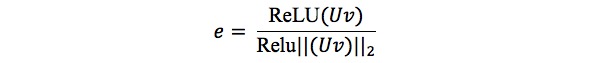


#### Concept Representations

Our model includes a component that learns representations for concepts and measures the similarity between an input phrase and the concepts by computing the dot product between these representations and the encoded phrase *e*.

We denote these representations by the matrix *H,* where each row corresponds to one concept. Our model does not learn *H* directly, but instead learns a matrix 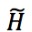
where each row 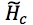
represents the features of concept *c* that are “novel” compared to its ancestors. Then, *H* can be derived by multiplying 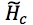
by the taxonomy’s ancestry matrix *A*: 
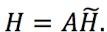


Each element of the ancestry matrix *A*_*i,j*_ is nonzero only if concept *j* is an ancestor of *i* (including *i*=*j*) and is calculated as: 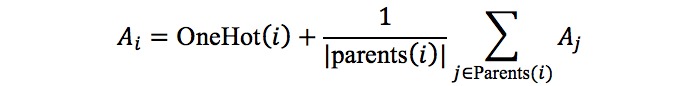


The final embedding of a concept would be the final embedding of its parent (or the average of its parents, in cases of multi-inheritance) plus its own raw embedding (ie, 
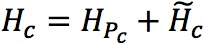
). In other words, the parent concept provides the global location in the embedding space, whereas the child concepts learn their local locations with respect to that space.

This has two major advantages. First, it incorporates the taxonomic structure as implicit prior information on the geometry of the concept embeddings. Second, by binding the embeddings of the concepts, training becomes more efficient, as for each concept, it is sufficient to learn only the local location with respect to its parent, rather than learning the absolute location from scratch. Furthermore, when the location of a concept gets updated, both its descendants and ancestors will also get updated, even if they do not have samples present in the mini-batch. More specifically, as a concept gets updated, the global locations provided to all its descendants are automatically updated as well, while the actual raw embedding of its ancestors will get updated through the backpropagation process. The results of our experiments quantitatively and qualitatively show the advantage of this approach in our task.

Finally, the classification is done by computing the dot product (plus a bias term) followed by a softmax layer as follows: 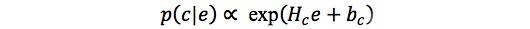


The taxonomy information can be ignored by setting *A* to the identity matrix *I*. In this scenario, the model would behave like an ordinary softmax classifier with the weight matrix 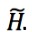


#### Training Procedure

Training is performed on the names and synonyms provided by the ontology. If a concept has multiple synonyms, each synonym-concept pair is considered as a separate training example. The parameters learned during the training are the encoder parameters *W* and *U*, and the concept representations through 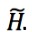
The fastText word vectors used in our experiments had a dimensionality of 100, while we set the dimensionality of the concept embeddings 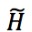
to be 1024. We used a filter size of 1024 for the convolution layer in the encoder, and the output of the dense layer used after the max-pooling layer was 1024. We trained our model by minimizing the cross-entropy loss between the softmax output and the class labels using Adam optimizer [35], with a learning rate of 0.002 and a batch size of 256. We trained our model for 100 epochs.

### Concept Recognition in a Sentence

To use our neural dictionary model to recognize concepts in a sentence or larger text, we extract all n-grams of one to seven words in the text and used the neural dictionary model to match each n-gram to a concept. We filter irrelevant n-grams by removing the candidates whose matching score (the softmax probability provided by the neural dictionary model) is lower than a threshold. This threshold is chosen based on the performance of the method (f-measure) on a validation set.

We also use random n-grams from an unrelated corpus (in our case Wikipedia) as negative examples labeled with a dummy *none* concept when training the neural dictionary model. This is done to reduce false positives that do not match to any concept (as opposed to false positives that are due to misclassification between two different concepts). To reduce the compute time, we made the assumption that phenotypic phrases have a length of at most 10 tokens, which we chose based on the empirical evidence that less than 0.8% of the names/synonyms in the HPO are longer than 10 tokens. As a result, the lengths of these n-grams were uniformly selected to be between 1 and 10.

After all the n-grams satisfying the conditions are captured, a postprocessing step is performed to ensure that the results are consistent. For every pair of overlapping captured n-grams, if both n-grams match the same concept, we retain the smaller n-gram. Otherwise, if they are matched to different concepts, we choose the longer n-gram, as this reduces the chances of choosing shorter general concepts in the presence of a more specific, longer, concept. For example, when annotating the sentence “The patient was diagnosed with conotruncal heart defect,” our method will favor choosing the longer, more specific concept “conotruncal heart defect” rather than the more general concept “heart defect.”

## Results

### Overview

To evaluate our model, we trained the model on the HPO and SNOMED-CT and applied it to a number of medical texts. We evaluated the model on two different tasks. In the first task, the model ranks concepts matching an input isolated phrase (synonym classification) and in the second task, concepts are recognized and classified from a document (concept recognition).

To assess the effectiveness of the techniques used in our model, we trained four variations of the model as follows:

- NCR: The full model, with the same architecture as described in the section Overview of the Neural Dictionary Model. The training data for this model includes negative examples.
- NCR-H: In this version, the model ignores the taxonomic relations by setting the ancestry matrix *A* to the identity matrix *I*.
- NCR-N: Similar to the original NCR, this version utilizes the taxonomic relations. However, this model has not been trained on negative samples.
- NCR-HN: A variation that ignores the taxonomy and has not been trained on negative examples.

To improve stability, we trained 10 different versions of our model, varying the random initialization of the model parameters and randomly reshuffling the training data across minibatches at the beginning of each training epoch. We created an ensemble of these 10 models by averaging their prediction probabilities for any given query and used this ensemble in all experiments.

### Data Sets

In most of our experiments, we used the HPO to train the neural dictionary model. To maintain consistency with previous work, we used the 2016 release of the HPO, which contains a total of 11,442 clinical phenotypic abnormalities seen in human disease and provides a total of 19,202 names and synonyms for them, yielding an average of 1.67 names per concept.

We evaluated the accuracy of our model trained on the HPO on two different data sets:

- PubMed: This data set contains 228 PubMed article abstracts, gathered and manually annotated with HPO concepts by Groza et al [14].
- Undiagnosed Diseases Program (UDP): This data set includes 39 clinical reports provided by National Health Institutes UDP [36]. Each case contains the medical history of a patient in unstructured text format and a list of phenotypic findings, recorded as a set of HPO concepts, gathered by the examining clinician from the patient encounter.

In order to examine the effectiveness of our model on different ontologies, we also trained the model on a subset of SNOMED-CT, which is a comprehensive collection of medical concepts that includes their synonyms and taxonomy. We evaluated the trained model for concept recognition using a subset of 2000 ICU discharge summaries from MIMIC-III. The discharge summaries are composed of unstructured text and are accompanied by a list of disease diagnosis terms in the form of ICD-9 codes.

Since SNOMED-CT provides a more sophisticated hierarchy than ICD-9 and a mapping between the two exists, we used a subset of SNOMED-CT concepts that include the ICD-9 concepts. We considered the 1292 most frequent ICD-9 concepts that have a minimum of 50 occurrences in MIMIC-III. These were filtered to 1134 concepts that also have at least one mapping SNOMED-CT concept, which were mapped to a total of 8405 SNOMED-CT concepts (more SNOMED-CT concepts because of one-to-many mappings). To have a single connected hierarchy of concepts, we also added all missing ancestors of these SNOMED-CT terms, resulting in a total of 11,551 SNOMED-CT concepts. To these additional 3146 SNOMED-CT concepts, we assigned the ICD-9 code mapped to the original SNOMED-CT term that had induced them (ie, their descendent). We trained NCR using these 11,551 SNOMED-CT concepts and the 21,550 names and synonyms associated with them.

### Synonym Classification Results

In this experiment, we evaluated our method’s performance in matching isolated phrases with ontology concepts. For this purpose, we extracted 607 unique phenotypic phrases that did not have an exact match among the names and synonyms in the HPO from the 228 annotated PubMed abstracts. We used our model to classify HPO concepts for these phrases and ranked them by their score.

In addition to the four variations of our model, we compared our method with one based on Apache Solr, customized to suggest HPO terms for phenotypic queries. This tool is currently in use as a component of the phenotyping software PhenoTips [16]. The results of this experiment are provided in Table 1. Since all the phrases in this data set are true phenotypic terms and PhenoTips reports at most 10 concepts for each phrase, we measured the fraction of the predictions where the correct label was among the top 1 (R@1) and top 5 (R@5) recalled concepts, instead of precision/recall. NCR outperformed PhenoTips by 20%-30% in this experiment. While NCR-N slightly outperformed regular NCR based on R@1, the experiments here contained no queries without phenotypic terms, which is the task that NCR-N was built to model.

An example phrase from this data set is “reduced retinal pigment,” labeled as HP:0007894. In our version of the HPO, there are four names/synonyms for this phrase: “hypopigmentation of the fundus,” “decreased retinal pigmentation,” “retinal depigmentation,” and “retinal hypopigmentation.” NCR correctly identified this concept as its top match. In contrast, the correct concept was not in the top 10 concepts reported by PhenoTips; the top reported concept was “retinal pigment epithelial mottling.”

**Table 1:** Synonym classification experiments on 607 phenotypic phrases extracted from 228 PubMed abstracts. Largest values for each category are italicized.

Method	Accuracy (%)	
	R@1^a^	R@5^b^
PhenoTips	28.9	49.3
NCR^c^	51.6	*80.6*
NCR-H^d^	45.5	69.8
NCR-N^e^	*55.8*	78.2
NCR-HN^f^	50.2	71.8

^a^R@1: recall using top 1 result from each method.

^b^R@5: recall using top 5 results from each method.

^c^NCR: Neural Concept Recognizer.

^d^NCR-H: variation of the NCR model that ignores taxonomic relations.

^e^NCR-N: variation of the NCR model that has not been trained on negative samples.

^f^NCR-HN: variation of the NCR model that ignores the taxonomy and has not been trained on negative examples.

### Concept Recognition Results

We evaluated the four versions of NCR for concept recognition and compared them with four rule-based methods: NCBO annotator [10], cTAKES [13], BioLarK [14], and OBO annotator [11]. The NCBO annotator is a general concept recognition tool with access to hundreds of biomedical ontologies, including the HPO. cTAKES is a more general medical knowledge extraction system primarily designed for SNOMED-CT, while BioLarK and the OBO annotator are concept recognizers primarily tailored for the HPO. Another method, called IHP (Identifying Human Phenotypes) [15], was recently introduced for identifying HPO terms in unstructured text using machine learning for named entity recognition and a rule-based approach for further extending them. However, this method is not directly comparable, as it only reports the text spans that are a phenotype and does not classify or rank matching HPO terms.

In order to choose a score threshold for filtering irrelevant concepts, we used 40 random PubMed abstracts as a validation set and compared the micro F1-score with different threshold values. The selected thresholds were 0.85, 0.8, 0.8, and 0.75 for NCR, NCR-H, NCR-N, and NCR-HN, respectively. Since the UDP data set contained fewer reports (39 in total), we did not choose a separate UDP validation set and used the same threshold determined for the PubMed abstracts. We tested our methods on the remaining 188 PubMed abstracts and the 39 UDP reports and calculated micro and macro versions of precision, recall, and F1-score, as shown in the following equations: 
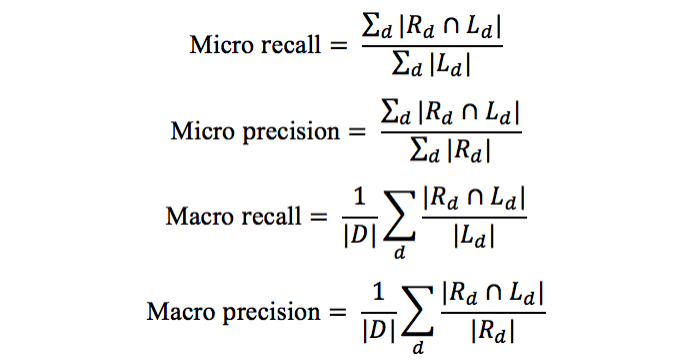


In these equations, *D* is the set of all documents and *R*_*d*_ and *L*_*d*_ notate the set of reported concepts and label concepts for the document *d*, respectively. In cases where ｜ *L*_*d*_｜ or ｜ *R*_*d*_｜were zero, we assigned a macro recall and macro precision of 1.0, respectively.

We also calculated a less strict version of accuracy measurements that takes the taxonomic relations of the concepts into consideration. For this, we extended the reported set and the label set for each document to include all their ancestor concepts, which we notate by *E(L*_*d*_*)* and *E(R*_*d*_*)*, respectively, and calculated an extended version of the precision and recall, as well as the Jaccard Index of the extended sets. The following equations show how these accuracies are derived: 
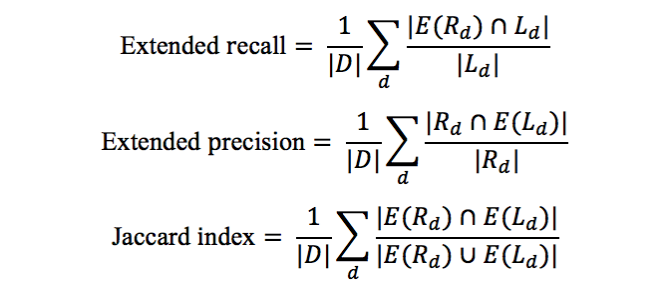


The measured micro and macro accuracies are provided in Tables 2 and 3 for the PubMed abstract and UDP data sets, respectively. The taxonomy-based extended accuracies and the Jaccard index results are available in Tables 4 and 5 for the abstracts and UDP data sets, respectively. In both experiments, based on the measurements of the Jaccard index and all three versions of micro, macro, and extended F1-scores, NCR had higher accuracy than all other baselines. Furthermore, by comparing the NCR and NCR-H, we observed that using the hierarchy information considerably improved the F1-score of the model in the abstract data set, although the F1-score of the UDP set was slightly lower. Finally, comparison of NCR and NCR-N showed that using negative examples during the training improved the overall accuracy for the abstract data set, while not using the negatives led to a narrow advantage with the UDP data set.

To verify the statistical significance of NCR’s superiority to the baselines, we aggregated both the abstract and UDP data sets for a total of 227 documents and calculated the F1-score for each document separately. This method is different from that used to calculate the F1-score presented in Tables 2-5, which only show a single measurement of F1-score per category. We compared the main version of NCR against BioLarK, which was our strongest baseline. NCR performed statistically significantly better (*P*=.003, Wilcoxon test).

To evaluate the effectiveness of the techniques employed in NCR on a different ontology, we trained the four variations of our model on the SNOMED-CT subset, using 200 MIMIC reports as the validation set and the remaining 1800 reports as a test set. We mapped each reported SNOMED-CT concept to the corresponding ICD-9 code and calculated the accuracy measurements (Table 6).

The results show that using the hierarchy information improved both micro and macro F1-scores. Since the labels were only available as ICD-9 codes, which do not hold a sufficiently rich hierarchical structure as opposed to HPO and SNOMED-CT, the Jaccard index and the extended accuracy measurements were less meaningful and were not calculated. We also ran the original cTAKES, which is optimized for SNOMED-CT concepts, on the 1800 test documents and filtered its reported SNOMED-CT results to ones that have a corresponding ICD-9. Although cTAKES had a high recall, the overall F1-scores were lower than those for NCR. Furthermore, using a method similar to the one used to calculate the statistical significance for the improvement relative to BioLark in the section above, we compared NCR with cTAKES and found that NCR performed statistically significantly better (*P*<.001, Wilcoxon test).

**Table 2:** Micro and macro measurements for concept recognition experiments on 188 PubMed abstracts. Neural Concept Recognizer models were trained on Human Phenotype Ontology. Largest values for each category are italicized.

Method	Micro (%)			Macro (%)		
	Precision	Recall	F1-score	Precision	Recall	F1-score
BioLarK	78.5	60.5	68.3	76.6	66.0	70.9
cTAKES^a^	72.2	55.6	62.8	74.0	61.4	67.1
OBO^b^	78.3	53.7	63.7	79.5	58.6	67.5
NCBO^c^	*81.6*	44.0	57.2	79.5	48.7	60.4
NCR^d^	80.3	62.4	*70.2*	*80.5*	68.2	*73.9*
NCR-H^e^	74.4	61.5	67.3	72.2	67.1	69.6
NCR-N^f^	78.1	*62.5*	69.4	76.6	*68.3*	72.2
NCR-HN^g^	77.1	57.2	65.7	76.5	63.4	69.3

^a^cTAKES: Clinical Text Analysis and Knowledge Extraction System.

^b^OBO: Open Biological and Biomedical Ontologies

^c^NCBO: National Center for Biomedical Ontology.

^d^NCR: Neural Concept Recognizer.

^e^NCR-H: variation of the NCR model that ignores taxonomic relations.

^f^NCR-N: variation of the NCR model that has not been trained on negative samples.

^g^NCR-HN: variation of the NCR model that ignores the taxonomy and has not been trained on negative examples.

**Table 3:** Micro and macro measurements for concept recognition experiments on 39 Undiagnosed Diseases Program clinical notes. Neural Concept Recognizer models were trained on Human Phenotype Ontology. Largest values for each category are italicized.

Method	Micro (%)			Macro (%)		
	Precision	Recall	F1-score	Precision	Recall	F1-score
BioLarK	27.6	21.0	23.9	28.7	21.6	24.6
cTAKES^a^	31.5	18.9	23.6	*37.5*	20.2	26.2
OBO^b^	26.8	20.5	23.2	28.8	20.1	23.7
NCBO^c^	*33.4*	16.9	22.5	37.1	19.9	25.9
NCR^d^	24.5	27.2	25.8	26.5	27.6	27.0
NCR-H^e^	25.1	26.8	25.9	26.2	27.0	26.6
NCR-N^f^	24.3	*28.5*	26.2	27.0	*28.9*	*27.9*
NCR-HN^g^	25.5	27.2	*26.4*	27.4	27.7	27.6

^a^cTAKES: Clinical Text Analysis and Knowledge Extraction System.

^b^OBO: Open Biological and Biomedical Ontologies

^c^NCBO: National Center for Biomedical Ontology.

^d^NCR: Neural Concept Recognizer.

^e^NCR-H: variation of the NCR model that ignores taxonomic relations.

^f^NCR-N: variation of the NCR model that has not been trained on negative samples.

^g^NCR-HN: variation of the NCR model that ignores the taxonomy and has not been trained on negative examples.

**Table 4:** Extended measurements for concept recognition experiments on 188 PubMed abstracts. Neural Concept Recognizer models were trained on Human Phenotype Ontology. Largest values for each category are italicized.

Method	Extended value (%)			Jaccard value (%)
	Precision	Recall	F1-score	
BioLarK	91.5	80.8	85.8	76.9
cTAKES^a^	95.6	73.9	83.3	72.1
OBO^b^	92.4	77.9	84.5	74.4
NCBO^c^	*95.8*	65.4	77.7	64.3
NCR^d^	93.3	82.1	*87.3*	*79.1*
NCR-H^e^	86.5	*83.8*	85.1	76.7
NCR-N^f^	90.6	83.1	86.7	78.2
NCR-HN^g^	89.7	78.9	83.9	73.2

^a^cTAKES: Clinical Text Analysis and Knowledge Extraction System.

^b^OBO: Open Biological and Biomedical Ontologies

^c^NCBO: National Center for Biomedical Ontology.

^d^NCR: Neural Concept Recognizer.

^e^NCR-H: variation of the NCR model that ignores taxonomic relations.

^f^NCR-N: variation of the NCR model that has not been trained on negative samples.

^g^NCR-HN: variation of the NCR model that ignores the taxonomy and has not been trained on negative examples.

**Table 5:** Extended measurements for concept recognition experiments on 39 Undiagnosed Diseases Program clinical notes. Neural Concept Recognizer models were trained on Human Phenotype Ontology. Largest values for each category are italicized.

Method	Extended value (%)			Jaccard index (%)
	Precision	Recall	F1-score	
BioLarK	58.9	42.6	49.5	29.5
cTAKES^a^	68.5	36.7	47.8	27.3
OBO^b^	59.2	46.4	52.0	31.3
NCBO^c^	*69.8*	37.2	48.5	27.2
NCR^d^	57.1	49.4	*53.0*	*31.5*
NCR-H^e^	54.0	49.4	51.6	30.5
NCR-N^f^	54.7	*50.5*	52.5	31.4
NCR-HN^g^	56.5	49.0	52.5	31.3

^a^cTAKES: Clinical Text Analysis and Knowledge Extraction System.

^b^OBO: Open Biological and Biomedical Ontologies

^c^NCBO: National Center for Biomedical Ontology.

^d^NCR: Neural Concept Recognizer.

^e^NCR-H: variation of the NCR model that ignores taxonomic relations.

^f^NCR-N: variation of the NCR model that has not been trained on negative samples.

^g^NCR-HN: variation of the NCR model that ignores the taxonomy and has not been trained on negative examples.

**Table 6:** Results for concept recognition experiments on 1800 Multiparameter Intelligent Monitoring in Intensive Care documents. The Neural Concept Recognizer models were trained on a subset of the Systematized Nomenclature of Medicine - Clinical Terms ontology. Largest values for each category are italicized.

Method	Micro (%)			Macro (%)		
	Precision	Recall	F1-score	Precision	Recall	F1-score
cTAKES^a^	9.1	*37.0*	14.6	8.7	*36.5*	14.1
NCR^b^	10.9	26.7	*15.5*	10.6	26.9	15.2
NCR-H^c^	10.0	30.6	15.1	9.6	30.4	14.6
NCR-N^d^	*11.2*	24.8	15.4	*11.1*	25.3	*15.4*
NCR-HN^e^	9.6	28.6	14.4	9.2	28.9	13.9

^a^cTAKES: Clinical Text Analysis and Knowledge Extraction System.

^b^NCR: Neural Concept Recognizer.

^c^NCR-H: variation of the NCR model that ignores taxonomic relations.

^d^NCR-N: variation of the NCR model that has not been trained on negative samples.

^e^NCR-HN: variation of the NCR model that ignores the taxonomy and has not been trained on negative examples.

### Qualitative Results

**Figure 2:**
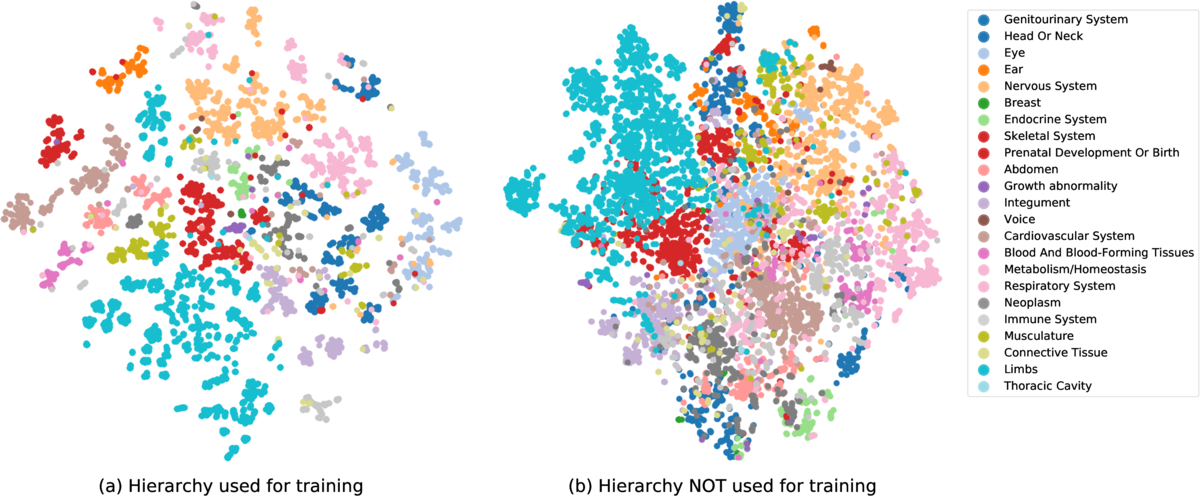
Visualization of the representations learned for Human Phenotype Ontology concepts. The representations are embedded into two dimensions using t-SNE. The colors denote the high-level ancestors of the concepts. The plot on the left shows the representations learned in NCR-N, where the taxonomy information was used in training, and the plot on the right shows representations learned for NCR-HN, where the taxonomy was ignored. NCR-HN: variation of the NCR model that ignores the taxonomy and has not been trained on negative examples; NCR-N: variation of the NCR model that has not been trained on negative samples; t-SNE: t-distributed stochastic neighbor embedding.

To better understand how utilizing the hierarchy information affects our model, we used t-SNE (t-distributed stochastic neighbor embedding) to embed and visualize the learned concept representations for the rows of matrix *H* for NCR-N (using hierarchy) and NCR-NH (not using the hierarchy), trained on the HPO. These representations are illustrated in Figure 2, where colors are assigned to concepts based on their high-level ancestor (the 23 children of the root). If a concept had multiple high-level ancestors, we chose one randomly. As is evident in the plots, the representations learned for NCR-N were better clustered than those for NCR-NH.

Interestingly, in the representations learned for NCR-N, concepts in categories that share children with many other categories, such as “Neoplasm” (dark grey), are located in the center of the plot, close to various other categories, while a category like “Abnormality of ear” (orange) forms its own cluster far from center and is separated from other categories.

To further investigate the false positives reported by NCR, we manually investigated the false positives reported by our method in three clinical reports randomly chosen from the UDP data set. We looked at false positives from the extended version of evaluations, which included concepts reported by our method, where neither the concepts nor any of their descendants were in the label set. This yielded a total number of 73 unique false positives for the three documents. Based on a manual analysis of these terms conducted by a medical expert on rare genetic diseases (coauthor DA), 47.9% of the reported false positives were actually correctly adding more information to the closest phenotype reported in the label set. One such example is “Congenital hypothyroidism on newborn screening.” Although our method correctly recognized “Congenital hypothyroidism,” the closest concept in the extended label set was “Abnormality of the endocrine system.” In an additional 8.2% of cases, our model correctly reported a more specific concept than that presented in the patient record, but the concept was sufficiently close to a specified phenotype for it not to be considered a novel finding. Furthermore, 16.4% of the reported false positives were, in fact, mentioned in the text, albeit as negations, such as “Group fiber atrophy was not seen.” In 6.8% of these cases, the reported phenotype was mentioned but not confidently diagnosed, such as “possible esophagitis and gastric outlet delay.”

## Discussion

### Principal Findings

Our experiments showed the high accuracy of NCR compared to the baselines in both synonym classification and concept recognition, where NCR consistently achieved higher F1-scores across different data sets. Furthermore, we showed that NCR’s use of the hierarchical information contributes to its higher performance.

In the synonym classification task, as evident in Table 1, all variations of NCR had a much better performance than the tool provided by PhenoTips. Furthermore, comparison of NCR and NCR-H showed that use of the hierarchy information considerably improved accuracy.

In concept recognition experiments, NCR had a better F1-score and Jaccard index than BioLarK and cTAKES on PubMed abstracts (Tables 2 and 4) and UDP reports (Tables 3 and 5). On both data sets, NCR had a higher recall, showing its ability to better generalize to synonymous terms that occurred in the text. In some experiments, NCBO achieved the highest precision; however, we should note that in the same experiments, NCR achieved a much better recall rate, and when taking both precision and recall into account, NCR had the highest F1-score.

Among different variations of NCR, use of the hierarchy information always led to a higher F1-score and Jaccard index. Having negative samples during training also generally improved accuracy; however, in some cases, this difference was small, and in some cases, NCR-N showed slightly better results.

Although the PubMed abstracts were manually annotated with HPO concepts by Groza et al [14], the text provided for UDP is not annotated and there is no explicit association between the provided HPO terms and phenotypic phrases in the text. However, since both the text and the terms referred to the same patients, a correspondence exists between them. This can explain the overall higher accuracy of all methods on PubMed data compared to UDP data. As a result, these performance measurements would be more meaningful when observed in a relative manner, which shows the better performance of NCR than the baselines.

The experiments on MIMIC data, where the model was trained on SNOMED-CT, resulted in a much lower accuracy than the two experiments performed using the HPO. In addition to the problem of implicit correspondence between labels and actual mentions in the text, in this experiment, we used a mapping between ICD-9 and SNOMED-CT terms, which can introduce further inconsistencies. On the other hand, for the sake of evaluating the techniques employed in our model on another ontology, use of the SNOMED-CT hierarchy, similar to the case with the HPO, improves the F1-scores (Table 3).

In addition to the quantitative results showing the advantage of using the hierarchy information, our visualization of the concept representations in Figure 2 shows that the representations learned for NCR-N are more cohesive compared to those for NCR-HN. Although in theory, NCR-N has the flexibility to learn representations identical to those of NCR-HN, the way our model utilizes the taxonomy connects the embedding of related concepts during training, which leads to better separated clusters.

NCR has already been used in several applications in practice. Currently, a version of NCR trained on the HPO is deployed as a component of PhenoTips software [16] and is being used in both annotation of clinical notes and term suggestion for manually entered phenotypes. Another example is PhenoLines [24], a software for visualizing disease subtypes, that relies on a mapping between HPO and Unified Medical Language System (UMLS) [37] terms. NCR was effectively used to help improve the coverage of their mapping. The code for NCR is available under the MIT license [38].

### Conclusions

In this paper, we presented a neural dictionary model that ranks matching concepts for a query phrase and can be used for concept recognition in larger text. Unlike other machine learning–based concept recognition tools, our training is solely performed on the ontology data (except the unsupervised learning of the word vectors) and does not require any annotated corpus. Another novelty of our model is our approach to using the taxonomic relations between concepts that, based on our experiments, improve synonym classification. Use of these taxonomic relations makes the training of our model easier by sharing knowledge between different concepts and providing implicit prior information on the similarity between concepts for the model. Furthermore, using multiple sources of information can improve the robustness of the model to potential errors in the input ontologies (eg, due to a mislabeled synonym).

NCR uses convolutional neural networks to encode query phrases into vector representations and computes their similarity to embeddings learned for ontology concepts. The model benefits from knowledge transfer between child and parent concepts by summing the raw embeddings of a concept’s ancestors to compute its final embedding. We tested our neural dictionary model by classifying 607 phenotypic phrases, and our model achieved a considerably higher accuracy than another method designed for this task and baseline versions of our model that do not use the taxonomy information. We also tested our method for concept recognition on full text using four data sets. In one setting, we trained our model on the HPO and tested it on two data sets, including 188 PubMed paper abstracts and 39 UDP clinical records, while in another setting, we trained the model on a subset of SNOMED-CT medical concepts and tested it on 1800 MIMIC ICU discharge notes. Our results showed the efficiency of our methods in both settings.

One major challenge for the concept recognition task is to filter candidates that do not match any class in the ontology. In our experiments, we approached this challenge by adding negative samples from Wikipedia in the training. Although this improved the results, it did not fully solve the problem, as there can be many relevant medical terms in a clinical text that are neither in an ontology nor available in any negative examples.

Although our experiments have shown the high accuracy of our model in classifying synonyms, we believe there is much more room for improvement in the overall concept recognition method, especially the way that n-grams are selected and filtered. Limitations of NCR include its relatively slower speed than several dictionary-based and rule-based methods and its limited ability to utilize contextual information for concept recognition. An interesting direction for future work is to investigate the possibility of using unsupervised methods for encoding phrases, such as skip-thought vectors [39] or the recently introduced language representation model BERT (Bidirectional Encoder Representations from Transformers) [40], to use the massive amount of available unannotated biomedical corpora for better generalization of classifying synonymous phrases and concept recognition.

## Abbreviations

BERT: Bidirectional Encoder Representations from Transformers
CRF: conditional random field
cTAKES: Clinical Text Analysis and Knowledge Extraction System
EHR: electronic health records
HPO: Human Phenotype Ontology
ICD-9: International Classification of Diseases - Ninth Revision
ICU: Intensive Care Unit
IHP: Identifying Human Phenotypes
LSTM: long short-term memory
MIMIC: Multiparameter Intelligent Monitoring in Intensive Care
NER: named entity recognizer
NCBO: National Center for Biomedical Ontology
NCR: Neural Concept Recognizer
NCR-H: variation of the NCR model that ignores taxonomic relations
NCR-HN: variation of the NCR model that ignores the taxonomy and has not been trained on negative examples
NCR-N: variation of the NCR model that has not been trained on negative samples
OBO: Open Biological and Biomedical Ontologies
R@1: recall using top 1 results from each method
R@5: recall using top 5 results from each method
ReLU: rectified linear unit
SNOMED-CT: Systematized Nomenclature of Medicine - Clinical Terms
t-SNE: t-distributed stochastic neighbor embedding
UDP: Undiagnosed Diseases Program
